# Draft Genome Sequence of *Pseudomonas* sp. Strain DrBHI1 (Phylum *Proteobacteria*)

**DOI:** 10.1128/genomeA.01090-17

**Published:** 2017-09-28

**Authors:** Alexandria K. Wilson, Virginia G. Watral, Michael L. Kent, Thomas J. Sharpton, Christopher A. Gaulke

**Affiliations:** a BioResource Research, Oregon State University, Corvallis, Oregon, USA; b Department of Microbiology, Oregon State University, Corvallis, Oregon, USA; c Department of Statistics, Oregon State University, Corvallis, Oregon, USA

## Abstract

Here, we report the draft genome sequence of *Pseudomonas* sp. strain DrBHI1. The total assembly length is 5,649,751 bp in 146 contigs. This strain was isolated from zebrafish (*Danio rerio*) feces.

## GENOME ANNOUNCEMENT

*Pseudomonas* is a large genus of Gram-negative, aerobic, rod-shaped bacteria known to inhabit a diversity of environmental and host-associated habitats (1, 2). Several species of this genus have been shown to be associated with the intestinal tracts of a variety of fish species (3), and some have been proposed as probiotic biocontrol agents in aquaculture (4).

*Pseudomonas* sp. strain DrBHI1 was isolated from the feces of zebrafish (*Danio rerio*). The isolation of this microbe was part of an undergraduate research project designed to increase the number of reference genomes from zebrafish-associated microbial communities represented in genome databases. Fecal pellets were collected from 9-liter tanks housing 12-month-old 5D line zebrafish using an aseptic technique. Fecal pellets were diluted, plated on brain heart infusion (BHI) agar, and incubated for 24 h at 27°C, and individual colonies were isolated. Isolates were inoculated in BHI broth and incubated overnight at 27°C. DNA was obtained using the UltraClean Microbial DNA isolation kit and the UltraClean PCR cleanup kit (Mo Bio). Universal 16S primers, 27F and 1492R (5), were used to PCR amplify the 16S rRNA gene, and an approximately 1,400-nucleotide product was gel purified using the UltraClean GelSpin DNA extraction kit (Mo Bio) and subsequently sequenced using an ABI 3730 DNA analyzer (Thermo Fisher Scientific). We then generated a list of full-length rRNA sequences closely related (>90% identity) to our isolate using BLAST (6) and the NCBI’s 16S rRNA database. These sequences were used to construct a maximum likelihood phylogeny using FastTree (7), which demonstrated that our isolate likely belonged to the genus *Pseudomonas*.

We next constructed a DNA library using the Nextera XT kit (Illumina), which we sequenced on an Illumina MiSeq platform. A total of 1,922,050 250-bp paired-end reads were generated, quality trimmed, filtered using ea-utils (8), and assembled using Velvet (9). The total assembly size was 5,649,751 bp in 146 contigs with an *N*_50_ value of 105,138 bp, average contig coverage of 125×, and 64.25% GC content. Genome completeness was quantified with PhyloSift (10), which approximated that this genome was >99% complete based on the presence of 37 universal single-copy genes. The genome was then annotated using the integrated microbial genomes (IMG) system (11) and predicted to contain 5,229 coding and 129 noncoding genes. Eight biosynthetic clusters were also identified (five nonribosomal peptide synthetase clusters, two bacteriocins, and one other cluster). To infer taxonomy, we used PhyloSift to place concatenated marker gene alignments on a reference phylogeny. This analysis suggested that our isolate was closely related to *Pseudomonas entomophila* L48 (https://figshare.com/articles/bhi1_concat_tree_pdf/5362594). However, the average nucleotide identity (ANI) (11) between our isolate and *P. entomophila* was low (89%). Given the discordance between the marker gene sequence placement and ANI, we were unable to classify this isolate beyond its genus and propose that it be classified *Pseudomonas* sp. strain DrBHI1.

### Accession number(s).

This whole-genome shotgun project has been deposited at DDBJ/ENA/GenBank under accession number NIBE00000000. The version described in this paper is the first version, NIBE01000000.

## References

[1] Anzai Y, Kim H, Park JY, Wakabayashi H, Oyaizu H 2000 Phylogenetic affiliation of the pseudomonads based on 16S rRNA sequence. Int J Syst Evol Microbiol 50:1563–1589. doi:10.1099/00207713-50-4-1563. 10939664

[2] Vodovar N, Vallenet D, Cruveiller S, Rouy Z, Barbe V, Acosta C, Cattolico L, Jubin C, Lajus A, Segurens B, Vacherie B, Wincker P, Weissenbach J, Lemaitre B, Médigue C, Boccard F 2006 Complete genome sequence of the entomopathogenic and metabolically versatile soil bacterium *Pseudomonas entomophila*. Nat Biotechnol 24:673–679. doi:10.1038/nbt1212. 16699499

[3] Llewellyn MS, Boutin S, Hoseinifar SH, Derome N 2014 Teleost microbiomes: the state of the art in their characterization, manipulation and importance in aquaculture and fisheries. Front Microbiol 5:207. doi:10.3389/fmicb.2014.00207. 24917852 PMC4040438

[4] Verschuere L, Rombaut G, Sorgeloos P, Verstraete W 2000 Probiotic bacteria as biological control agents in aquaculture. Microbiol Mol Biol Rev 64:655–671. doi:10.1128/MMBR.64.4.655-671.2000. 11104813 PMC99008

[5] Jiang H, Dong H, Zhang G, Yu B, Chapman LR, Fields MW 2006 Microbial diversity in water and sediment of Lake Chaka, an athalassohaline lake in northwestern China. Appl Environ Microbiol 72:3832–3845. doi:10.1128/AEM.02869-05. 16751487 PMC1489620

[6] Altschul SF, Gish W, Miller W, Myers EW, Lipman DJ 1990 Basic local alignment search tool. J Mol Biol 215:403–410. doi:10.1016/S0022-2836(05)80360-2. 2231712

[7] Price MN, Dehal PS, Arkin AP 2010 FastTree 2—approximately maximum-likelihood trees for large alignments. PLoS One 5:e9490. doi:10.1371/journal.pone.0009490. 20224823 PMC2835736

[8] Aronesty E 2013 Comparison of sequencing utility programs. Open Bioinform J 7:1–8. doi:10.2174/1875036201307010001.

[9] Zerbino DR, Birney E 2008 Velvet: algorithms for de novo short read assembly using de Bruijn graphs. Genome Res 18:821–829. doi:10.1101/gr.074492.107. 18349386 PMC2336801

[10] Darling AE, Jospin G, Lowe E, Matsen FA, Bik HM, Eisen JA 2014 PhyloSift: phylogenetic analysis of genomes and metagenomes. PeerJ 2:e243. doi:10.7717/peerj.243. 24482762 PMC3897386

[11] Huntemann M, Ivanova NN, Mavromatis K, Tripp HJ, Paez-Espino D, Palaniappan K, Szeto E, Pillay M, Chen IM, Pati A, Nielsen T, Markowitz VM, Kyrpides NC 2016 Erratum to: the standard operating procedure of the DOE-JGI Microbial Genome Annotation Pipeline (MGAP v.4). Stand Genomic Sci 11:27. doi:10.1186/s40793-016-0148-8. 27004084 PMC4800771

